# Preliminary Observations of the Loke Microdialysis in an Experimental Pig Model: Are We Ready for Continuous Monitoring of Brain Energy Metabolism?

**DOI:** 10.1007/s12028-024-02080-5

**Published:** 2024-07-31

**Authors:** Teodor Svedung Wettervik, Anders Hånell, Kerstin M. Ahlgren, Lars Hillered, Anders Lewén

**Affiliations:** 1 Department of Medical Sciences, Section of Neurosurgery, Uppsala University, 751 85 Uppsala, Sweden; 2 Department of Surgical Sciences, Uppsala University, 751 85 Uppsala, Sweden

**Keywords:** Brain injury, Energy metabolism, Loke, Microdialysis, Neurointensive care

## Abstract

**Background:**

Brain energy metabolism is often disturbed after acute brain injuries. Current neuromonitoring methods with cerebral microdialysis (CMD) are based on intermittent measurements (1–4 times/h), but such a low frequency could miss transient but important events. The solution may be the recently developed Loke microdialysis (MD), which provides high-frequency data of glucose and lactate. Before clinical implementation, the reliability and stability of Loke remain to be determined in vivo. The purpose of this study was to validate Loke MD in relation to the standard intermittent CMD method.

**Methods:**

Four pigs aged 2–3 months were included. They received two adjacent CMD catheters, one for standard intermittent assessments and one for continuous (Loke MD) assessments of glucose and lactate. The standard CMD was measured every 15 min. Continuous Loke MD was sampled every 2–3 s and was averaged over corresponding 15-min intervals for the statistical comparisons with standard CMD. Intravenous glucose injections and intracranial hypertension by inflation of an intracranial epidural balloon were performed to induce variations in intracranial pressure, cerebral perfusion pressure, and systemic and cerebral glucose and lactate levels.

**Results:**

In a linear mixed-effect model of standard CMD glucose (mM), there was a fixed effect value (± standard error [SE]) at 0.94 ± 0.07 (*p* < 0.001) for Loke MD glucose (mM), with an intercept at − 0.19 ± 0.15 (*p* = 0.20). The model showed a conditional *R*^2^ at 0.81 and a marginal *R*^2^ at 0.72. In a linear mixed-effect model of standard CMD lactate (mM), there was a fixed effect value (± SE) at 0.41 ± 0.16 (*p* = 0.01) for Loke MD lactate (mM), with an intercept at 0.33 ± 0.21 (*p* = 0.25). The model showed a conditional *R*^2^ at 0.47 and marginal *R*^2^ at 0.17.

**Conclusions:**

The established standard CMD glucose thresholds may be used as for Loke MD with some caution, but this should be avoided for lactate.

**Supplementary Information:**

The online version contains supplementary material available at 10.1007/s12028-024-02080-5.

## Introduction

Cerebral microdialysis (CMD) is a focal monitoring method of neurochemistry [1, 2]. The catheter is inserted into the brain parenchyma and is perfused with artificial cerebrospinal fluid at a rate between 0.3 and 2.0 µL/min [1–6]. The fluid in the catheter takes up metabolites by means of diffusion from the cerebral interstitial space over a semipermeable membrane. The fluid is then collected in vials and intermittently quantified (1–4 times/h). The method was developed in the 1970s [7] and introduced in the neurointensive care (NIC) unit in the early 1990s [5]. In the NIC setting, CMD has particularly been used to study the dynamics of brain energy metabolism and has proven to be useful for many clinical purposes [1]. First, too low/high CMD glucose levels and a high lactate-to-pyruvate ratio (LPR) have been associated with unfavorable outcome after acute brain injury and are therefore of clinical interest as potential treatment targets [8–10]. Second, CMD glucose levels decrease and the LPR increases in response to cerebral ischemia [9, 11–13]. Because many patients with acute brain injury develop autoregulatory disturbances, cerebral ischemia may occur despite adequate cerebral perfusion pressure (CPP) levels [14]. Thus, these CMD variables, rather than standardized, fixed CPP levels, may be better surrogate measures for the individualized lower CPP threshold when cerebral decompensation starts to occur because of ischemia [15, 16]. Third, CMD has been helpful in characterizing nonperfusion-related energy metabolic disturbances [15–17]. Consequently, when used together with other multimodal monitoring tools during NIC of patients with acute brain injury, CMD may be one important adjunct to determine specific pathophysiological mechanisms. For example, in cases with normal CPP, cerebral blood flow, brain tissue oxygenation, and cerebral energy supply (glucose and pyruvate) but an elevated LPR, the combined interpretation would be energy failure due to mitochondrial dysfunction [17, 18]. Ultimately, these energy metabolic characterizations may be useful for setting the indication for targeted therapy in the near future as certain agents such as cyclosporin emerge as a promising treatment for mitochondrial dysfunction [18]. Fourth, CMD energy metabolic variables may add important prognostic information, as they have been strongly linked with focal brain tissue survival [19] and long-term functional outcome [9, 11, 12].

However, despite these beneficial aspects of CMD, it has remained a promising multimodality monitoring tool in the NIC unit for more than 30 years, but it has yet to become implemented in the standard of care in international guidelines for severe acute brain injury [20, 21]. One particular challenge with CMD is the low-frequency sampling (typically 1 time/h), which significantly decreases its sensitivity to detect transient but important events. For example, if a patient exhibited a significant ischemia for 10 min that led to permanent brain damage, but there was otherwise normal to hyperemic blood flow during that hour, the CMD variables for the corresponding sampling period were most likely only slightly disturbed because of dilution. To overcome this issue, high-frequency microdialysis (MD) (MD system, branded as “Loke”) was developed, providing approximately 0.5-Hz data of glucose and lactate using biosensors, and has emerged as an attractive solution. However, before clinical implementation of this new device, there is a need to validate it in vivo in relation to the traditional intermittent CMD. In this way, we can determine whether previously established glucose (e.g., < 0.2 mM) and lactate (e.g., > 4 mM) thresholds also apply for this new technique [8]. In addition, it is important to determine the stability of these variables in vivo to appreciate the signal-to-noise ratio. Therefore, we conducted this experimental pig study, in which we induced systemic and cerebral glucose and lactate variations and investigated the stability of the Loke MD and the correlation of glucose and lactate levels obtained with simultaneous standard CMD.

## Methods

### Animals and Ethic Statements

We included four pigs (*Sus scrofa domesticus*, crossbreed; Norwegian Landrace [1/4], Yorkshire [1/4], and Hampshire [1/2], 3 females and 1 male) aged 2–3 months (Supplementary Table 1). After premedication and induction of anesthesia, the animals received continuous intravenous analgesia and were under deep anesthesia throughout the experiments. All efforts were made to minimize suffering. The study was approved by the Animal Ethics Committee in Uppsala, Sweden (Dnr 5.8.18-21799/2022), and performed at the Hedenstierna laboratory, Uppsala University, Sweden. All applicable institutional and/or national guidelines for the care and use of animals were followed.

### Anesthesia and Mechanical Ventilation

The pigs were premedicated with Zoletil Forte (tiletamine/zolazepam) at 6 mg/kg (Virbac, Kolding, Denmark) and Rompun (xylazine) at 2.2 mg/kg (Elanco Denmark Aps, Ballerup, Denmark) and given a bolus of fentanyl at 5 μg/kg (Braun, Danderyd, Sweden) when intravenous access was established. Anesthesia was maintained with ketamine (Abcur, Helsingborg, Sweden) at 30 mg/kg/h, fentanyl (Braun, Danderyd, Sweden) at 4 μg/kg/h, and midazolam (Accord Healthcare, Solna, Sweden) at 0.12 mg/kg/h during the whole experiment. After adequate levels of anesthesia and analgesia were ascertained by the absence of reaction to pain stimulus between the rear hooves, rocuronium (Braun, Kista, Sweden) at 2.5 mg/kg/h was infused intravenously as a muscle relaxant. Ringer acetate (Baxter, Kista, Sweden) was infused intravenously at a rate of 10 mL/kg/h during the first hour and thereafter at a rate of 5 mL/kg/h. Animals were tracheostomized and mechanically ventilated (Servo I, Maquet, Solna, Sweden).

### Study Design

The standard CMD and Loke MD were inserted in the right frontal lobe adjacent to each other. The arterial blood pressure and arterial blood gas (ABG) were monitored via an arterial line inserted in the right carotid artery. A pulmonary artery catheter and a triple lumen central venous catheter were inserted via the right jugular vein and used for monitoring the animals and for fluid infusions. Furthermore, an intracranial balloon was inserted in the epidural space via a contralateral burr hole. This study on Loke was a part of a larger project on the role of hyperglycemia in the “healthy” and “injured” brain. Thus, the pigs also received monitors of intracranial pressure, focal cerebral blood flow, and brain tissue oxygenation. This study solely focused on validation of Loke MD vs. standard CMD, whereas the analyses of the interactions between cerebral physiological variables will be investigated in a separate study in a larger cohort of pigs.

To evaluate the correlation between standard CMD and Loke MD for glucose and lactate, we induced changes in systemic (hyperglycemia) and cerebral physiology (intracranial hypertension with cerebral hypoperfusion) to increase/decrease glucose delivery and to alter cerebral energy metabolism [22, 23]. First, to increase arterial glucose content and thereby the cerebral glucose delivery, a moderate (0.4 g/kg) bolus dose of glucose was injected intravenously. Second, after 30 min, another, more significant (1.0 g/kg) bolus dose of glucose was injected. Third, 30 min after the second glucose injection, an intravenous insulin dose (10 units) was given to restore normoglycemia. Fourth, after arterial glucose normalization, the intracranial balloon was inflated with a few mL of water to induce intracranial hypertension and reduce cerebral blood flow and glucose delivery. The balloon volume was set to target a CPP around 30 mm Hg, which in previous studies, has appeared to be sufficient to significantly impair cerebral energy metabolism [23]. The CPP target was also adjusted individually for each pig to make sure that focal cerebral blood flow and brain tissue oxygenation also deteriorated. Fifth, after 30 min of balloon inflation, a bolus dose (1.4 g/kg) of glucose was injected. Sixth, after another 30 min, insulin (10 units) was administered intravenously to restore arterial glucose, and then the experiment was terminated.

### Monitors, Data Acquisition, and Analysis

The pigs were monitored with repeated ABG via an arterial line. Two CMD catheters were inserted with the tip in a gyrus in the right frontal lobe via two separate, adjacent burr holes (Supplementary Fig. 1). For the standard CMD, the 71 High Cut-Off Brain MD catheter with a membrane length of 10 mm and a membrane cutoff of 100 kDa (M Dialysis AB, Stockholm, Sweden) was used. The inner diameter of the inlet and outlet tubing was 0.15 mm. The catheters were perfused by means of a microinjection pump with a rate at 2 µL/min using custom-made sterile artificial cerebrospinal fluid (NaCl 147 mmol/L [mM], KCl 2.7 mM, CaCl_2_ 1.2 mM, MgCl_2_ 0.85 mM, and 1.5% human albumin). The fluid was collected in vials every 15 min and immediately put in a − 20 °C freezer. Cerebral glucose and lactate from these vials were later estimated using an ISCUSflex Microdialysis Analyzer (M Dialysis AB). Every vial was time stamped in relation to the aforementioned experimental steps.

For the Loke MD, the CMA 70 Brain MD catheter with a membrane length of 10 mm and a membrane cutoff of 20 kDa (M Dialysis AB, Stockholm, Sweden) was used. The inner diameter of the inlet and outlet tubing was 0.15 mm. The catheter was perfused by means of a microinjection pump within the Loke system at the same rate as the standard CMD (2 µL/min) using custom-made sterile artificial cerebrospinal fluid (NaCl 147 mmol/L [mM], KCl 2.7 mM, CaCl_2_ 1.2 mM, and MgCl_2_ 0.85 mM). Cerebral glucose and lactate were continuously analyzed via electrochemical biosensors (approximately 0.5 Hz), and the estimations were immediately displayed on the Loke screen. The biosensors contained multiple electrodes. Each electrode had three layers (catalase membrane [preventing crosstalk between electrodes], diffusion limiting membrane [determining the sensitivity for each substrate], and oxidase membrane [substrate-specific enzymatic oxidation to H_2_O_2_]). In turn, H_2_O_2_ reacted with the underlying platinum electrode, which produced an electric current that was proportional to the substrate concentration. Before implantation, the Loke system had been flushed and calibrated for approximately 2 h. The Loke system possessed occasional automatic reflushes during the experiments, which led to some gaps in the data.

An important issue for the data analysis was to determine which time intervals that the standard CMD and Loke MD data reflected to more accurately decide the strength of the correlation between their glucose and lactate estimations. Particularly, there was a difference in delay time (3 min [standard] and 11 min [Loke]) from the membrane to the vial and sensor, respectively, due to differences in the catheter length and volume for each MD system. Thus, we adjusted the time stamps for each MD to reflect when the fluid had passed the membrane (− 3 min [standard] and − 11 min [Loke] from the real time [ABG reference]). Furthermore, for the purposes of statistical analysis, the Loke estimations of glucose and lactate were averaged over the corresponding 15-min interval (after the time adjustment previously described) as the intermittent standard CMD measurement.

### Statistical Analysis

Statistical analyses were conducted in RStudio software (version 2022.12.0) [24]. The association between standard CMD and Loke MD for the energy metabolites glucose and lactate was evaluated with linear mixed-effect models. Standard CMD glucose or lactate was used as dependent variable, whereas Loke MD glucose or lactate was used as a fixed effect, and the pig (one to four) was used as a random effect. The association between standard CMD and Loke MD glucose/lactate was visualized using temporal trendlines for each pig, scatter plots, and Bland–Altman plots. When calculating the root mean squared error (RMSE), the error was defined as the difference between standard CMD and Loke MD and the mean calculated either for all samples from a given time point or from a given subject. Furthermore, to estimate the stability in Loke estimations of glucose and lactate, the absolute difference between two adjacent values (2–3 s apart) and the absolute difference between the maximal and minimal value during 1 min were visualized for these metabolites in histograms. A *p* value < 0.05 was considered statistically significant.

## Results

### Standard CMD Versus Loke MD: Glucose

**Fig. 1:**
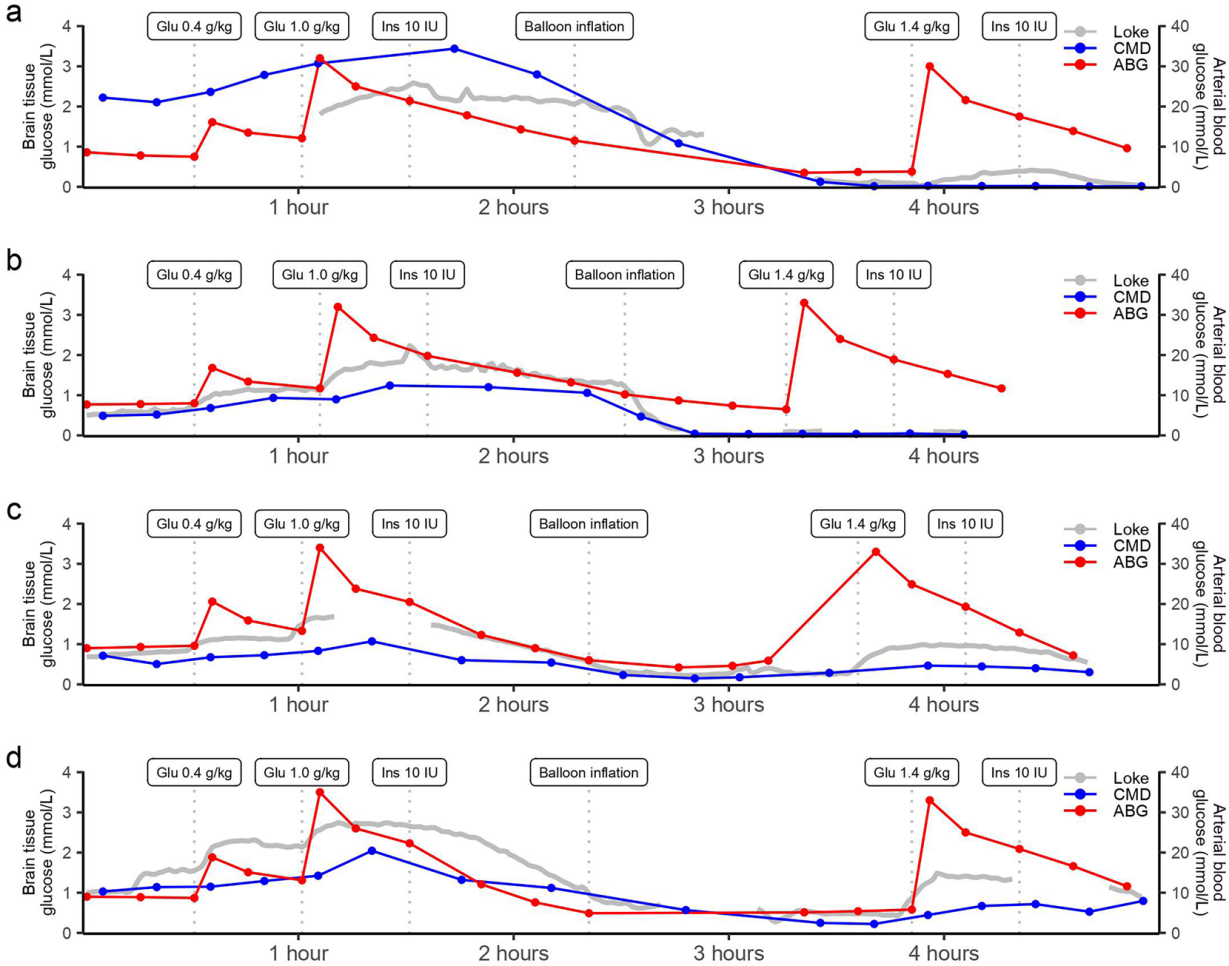
Temporal dynamics of arterial and cerebral (standard and Loke MD) glucose during the experiment for each of the four pigs. **a** Pig 1. **b** Pig 2. **c** Pig 3. **d** Pig 4. During the experiment, arterial hyperglycemia was induced by means of two bolus doses of intravenous glucose, which was followed by intravenous insulin administration to induce normoglycemia. Then a brain injury with intracranial hypertension with an inflated intracranial balloon was induced, which was again followed by injection of an intravenous bolus dose of glucose and then intravenous insulin again to restore arterial glucose. As indicated, both standard cerebral and Loke cerebral glucose levels increased during hyperglycemia, decreased with insulin and after induction of intracranial hypertension, but then increased again with hyperglycemia, except in **a** and **b** when the glucose levels had become completely depleted before the hyperglycemia episode. Thus, increased arterial glucose content had less impact on cerebral glucose delivery and tissue levels when the perfusion was most likely very limited. As outlined, the increase in cerebral glucose after intravenous glucose was smaller in case of intracranial hypertension as compared to a “healthy” state. There were some gaps in the Loke MD data due to system reflush. ABG arterial blood gas, CMD cerebral microdialysis, MD microdialysis

**Fig. 2:**
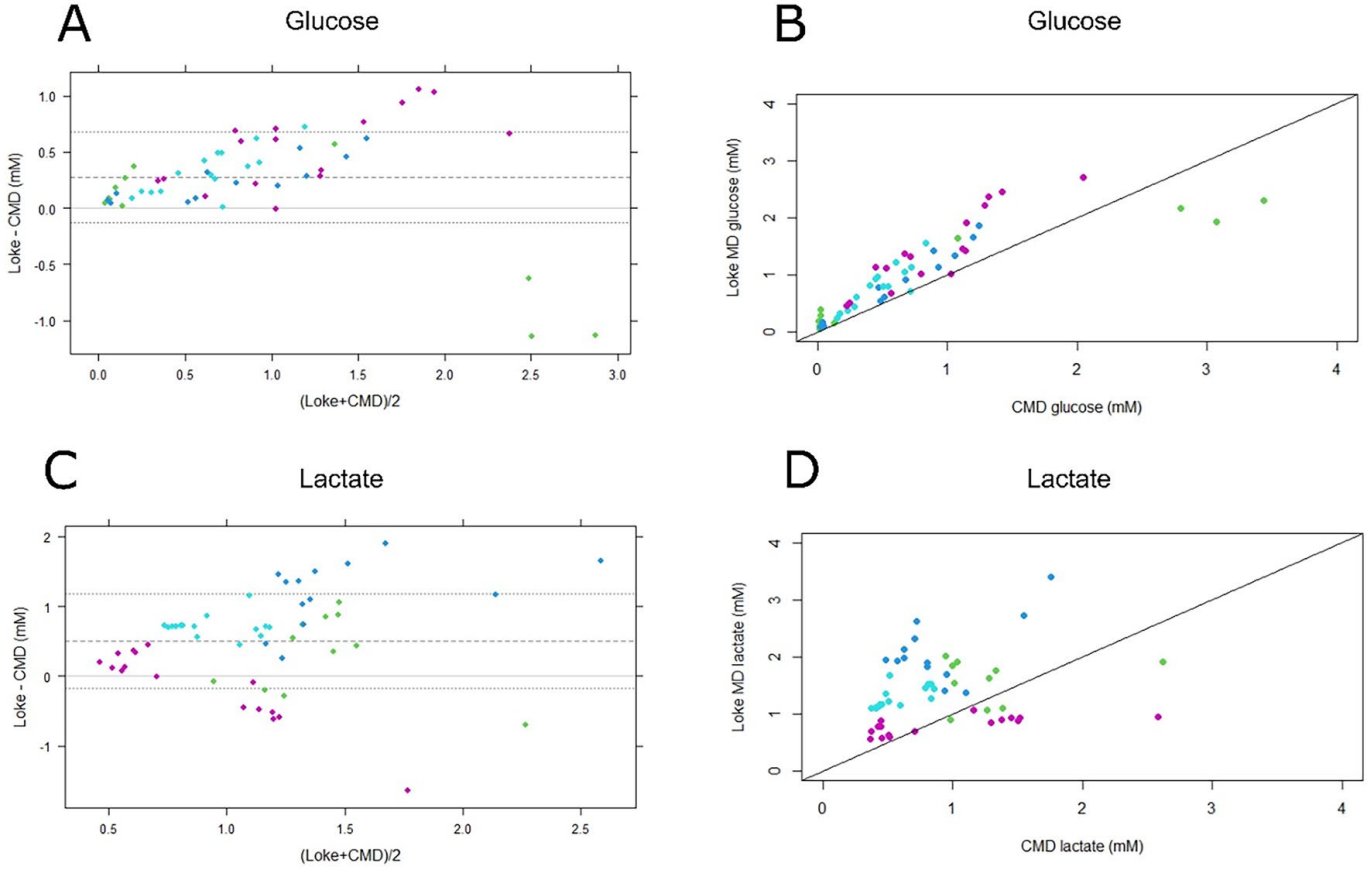
Strong associations between glucose, but not lactate, CMD and Loke MD estimations: Bland–Altman plot and scatter plot analysis of glucose and lactate. **a** and **b** A Bland–Altman plot and a scatter plot of glucose (mean ± SD 0.27 ± 0.41) estimations from standard CMD and Loke MD. As indicated, the difference between these was smaller for lower glucose values and greater for higher glucose values. The dots were colored differently for each of the four pigs. **c** and **d** A Bland–Altman plot (mean ± SD 0.50 ± 0.68) and a scatter plot of lactate estimations from standard CMD and Loke MD. As indicated, the variation between the two different modalities were greater for lactate than for glucose. CMD cerebral microdialysis, MD microdialysis

**Fig. 3:**
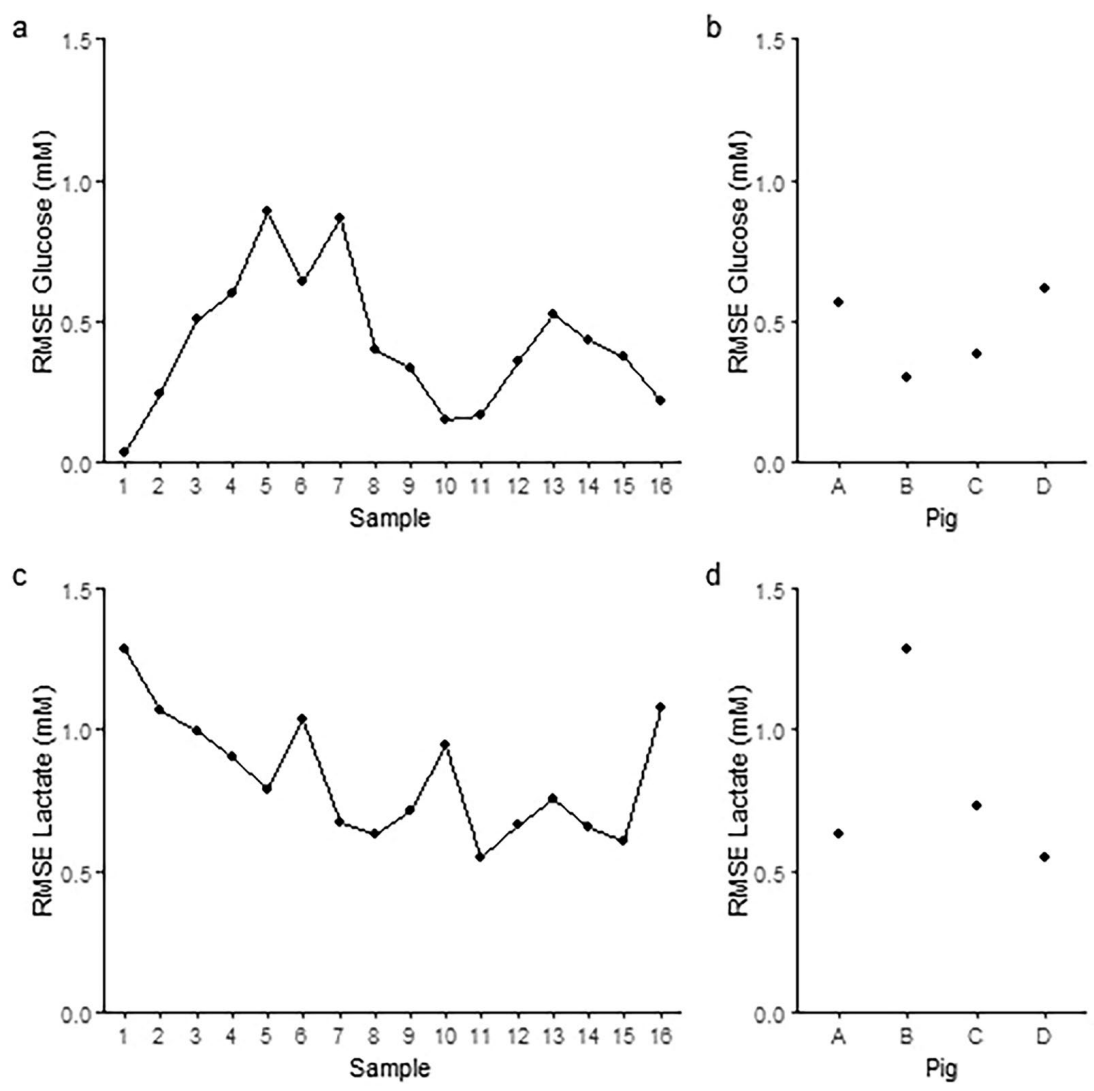
RMSE across samples and subjects. The difference between standard CMD and Loke MD expressed as RMSE for glucose (**a** and **b**) and lactate (**c** and **d**) across samples (**a** and **c**) and subjects (**b** and **d**). CMD cerebral microdialysis, MD microdialysis, RMSE root mean squared error

As outlined in the trendlines of standard CMD and Loke MD (Fig. 1), glucose levels increased in response to induced hyperglycemia, decreased after insulin treatment, decreased further during intracranial hypertension, and increased again after another episode of hyperglycemia. Although the absolute cerebral glucose level differed slightly between standard CMD and Loke MD, the temporal changes were comparable. As outlined in the scatter plot and the Bland–Altman plot (Fig. 2), the absolute difference between standard CMD and Loke MD was small except for higher glucose values above 1.5 mM. Figure 3 also demonstrates a relatively low RMSE over time and for each pig. In line with these findings (Table 1), a linear mixed-effect model of standard MD glucose (mM) demonstrated a fixed effect value (± standard error [SE]) at 0.94 ± 0.07 (*p* < 0.001) for Loke MD glucose (mM), with an intercept at − 0.19 ± 0.15 (*p* = 0.20). The model exhibited a conditional *R*^2^ at 0.81 and a marginal *R*^2^ at 0.72, that is, the fixed effect (absolute glucose value) variable explained the variation of the model to a large extent, whereas the random effect (pigs) only added a small explanatory value.

**Table 1:** Association between Loke microdialysis and standard CMD estimations of glucose and lactate

Variable	Value	SE	*p* value
Standard CMD glucose			
Intercept	− 0.19	0.15	0.20
Loke glucose	0.94	0.07	< 0.001
Standard CMD lactate			
Intercept	0.33	0.28	0.25
Loke lactate	0.41	0.16	0.01

Random factors: pig (one to four). Glucose model, Akaike information criteria = 60, conditional *R*^2^ = 0.81, and marginal *R*^2^ = 0.72. Lactate model, Akaike information criteria = 81, conditional *R*^2^ = 0.47, and marginal *R*^2^ = 0.17

*CMD* cerebral microdialysis

### Standard CMD Versus Loke MD: Lactate

**Fig. 4:**
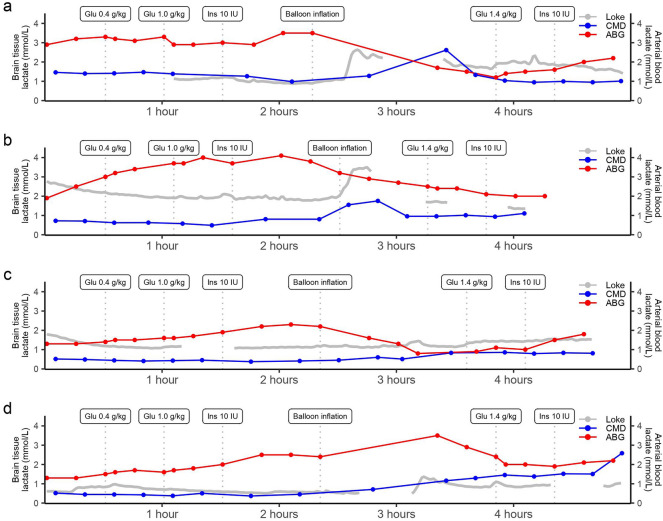
Temporal dynamics of arterial and cerebral (standard and Loke MD) lactate during the experiment for each of the four pigs. **a** Pig 1. **b** Pig 2. **c** Pig 3. **d** Pig 4. During the experiment, arterial hyperglycemia was induced by means of two bolus doses of intravenous glucose, which was followed by intravenous insulin administration to induce normoglycemia. Then a brain injury with intracranial hypertension with an inflated intracranial balloon was induced, which was again followed by injection of an intravenous bolus dose of glucose and then intravenous insulin again to restore arterial glucose. As indicated, there was only a limited change in standard CMD and Loke MD lactate levels in response to the initial hyperglycemia trial; however, cerebral lactate levels increased after the induction of intracranial hypertension. There were some gaps in the Loke MD data due to reflush. ABG arterial blood gas, CMD cerebral microdialysis, MD microdialysis

As outlined in the trendlines of standard CMD and Loke MD (Fig. 4), lactate levels were stable or increased slightly in response to induced hyperglycemia. There was a more pronounced increase in cerebral lactate levels during intracranial hypertension. Figure 3 also demonstrates a moderate RMSE over time and for each pig. In a linear mixed-effect model of standard CMD lactate (mM; Table 1), the fixed effect value (± SE) was 0.41 ± 0.16 (*p* = 0.01) for Loke MD lactate (mM), with an intercept at 0.33 ± 0.21 (*p* = 0.25). The model showed a conditional *R*^2^ at 0.47 and a marginal *R*^2^ at 0.17, that is, the fixed effect (absolute MD lactate value) only explained a small part of the variation, whereas the added value of the random effect had a strong effect on this variation.

### Loke Cerebral MD: Variability in Glucose and Lactate Estimations

**Fig. 5:**
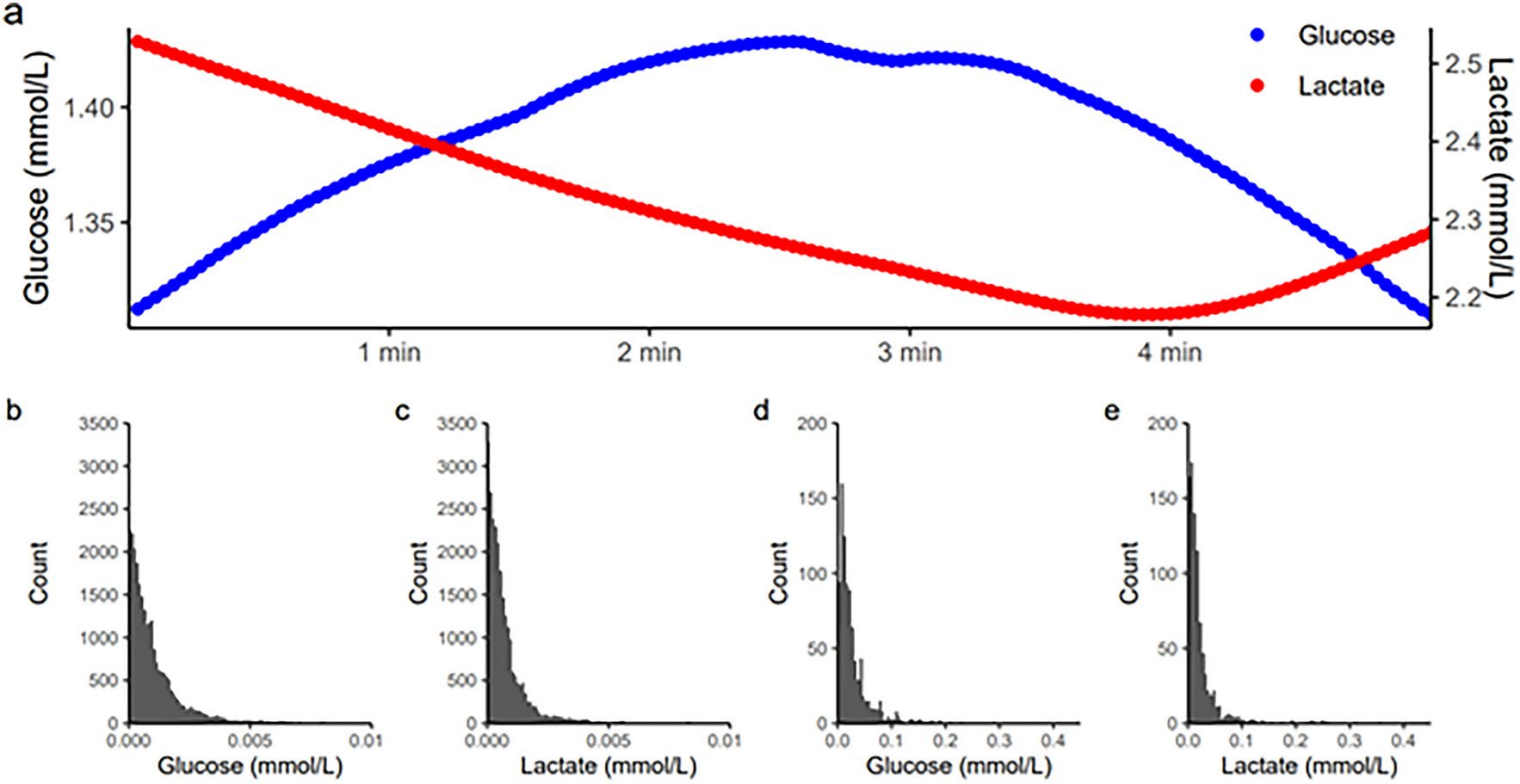
Variability in value-by-value and within-minute values of Loke microdialysis glucose and cerebral microdialysis lactate. **a** Value-by-value measures of glucose and lactate over 5 min. **b** and **c** Histograms of the absolute difference (mM) value-by-value for glucose and lactate. **d** and **e** Histograms of the absolute difference (mM) between maximal and minimal values within 1 min for glucose and lactate

For glucose and lactate according to Loke (Fig. 5), the absolute difference, value-by-value, was usually < 0.01 mM and the absolute difference in maximal and minimal glucose value within 1 min rarely exceeded 0.2 mM.

## Discussion

In this experimental pig study, we were able to validate that the glucose estimations from continuous Loke MD were comparable to the traditional intermittent CMD estimations. Thus, we suggest that a similar threshold as has been developed for the traditional intermittent CMD can be translated to the Loke system. However, although there was a significant linear association between the lactate estimations for Loke MD and those for traditional CMD, the correlation was weak. This fact could be explained by technical, analytical aspects or by a cerebral spatial variation in cerebral lactate. However, it calls into question whether the established thresholds of CMD lactate can be used for Loke MD. Overall, Loke displayed a very small value-to-value variability for both glucose and lactate, indicating that the biosensors were stable with a high signal-to-noise ratio in vivo.

First, the linear correlation between Loke and standard CMD was strong for glucose. Most measurements were close to 1:1; however, the variation tended to be larger for high values > 1.5 mM. This finding supports that Loke MD can be used in vivo to monitor cerebral glucose and that similar thresholds as has been elucidated for the standard CMD can be applied for treatment indications and outcome prognosis [8]. The absolute value-by-value (< 0.01 mM) and within-minute variation (< 0.2 mM) in Loke MD glucose was typically small. Because the induced changes in systemic and cerebral physiology were sustained over longer periods, such a small short-term variability was expected. Thus, Loke MD appears to exhibit a high signal-to-noise ratio. This finding is important because it opens the door for online detection and explorations of brief and transient pathophysiological events with energy metabolic disturbances, including cortical spreading depolarizations [25]. Such events need to be further investigated using Loke MD and in relation to electrophysiology in future studies. It would also be highly interesting to study the short-term variability in energy metabolism in relation to CPP and autoregulatory impairment in patients at risk of ischemia, such as in the vasospasm phase after aneurysmal subarachnoid hemorrhage.

Second, there was a linear correlation between Loke MD and standard CMD lactate, but it was overall weak. The absolute values differed, and the fixed effect was 0.4:1 (i.e., far from 1:1). One explanation could be that the MD catheters were inserted through two separate burr holes and that the spatial variation in cerebral lactate is larger than that for glucose. However, based on previous imaging studies, the spatial lactate variation in nearby tissue appears low [26]. Furthermore, the intracranial pressure insult was induced by inflation of a contralateral balloon, and the distance between the balloon and each of the MD catheters was negligible and therefore most likely inflicted an equal insult to the focal brain tissue surrounding each catheter. Another explanation could be that the standard cerebral MD vials were put in a freezer for storage during the experiments and were analyzed later. However, previous studies on glucose and lactate from the cerebrospinal fluid [27, 28] and CMD [29] indicate that the concentration of both of these metabolites should remain stable if they are immediately stored in a freezer until analysis. A third explanation could be that there is in fact a significant margin of error between the Loke sensor and the ISCUSflex Microdialysis Analyzer. Altogether, because of the weakness in the correlation and differences in absolute values between the standard CMD and Loke MD, this calls into questions if previously established lactate thresholds [8] can be used for Loke MD in clinical practice.

Third, an important general note for this study is that we used a high perfusion rate at 2.0 µL/min rather than the clinical standard at 0.3 µL/min because we aimed to decrease delay time due to time constraints for this experiment. The higher perfusion rate decreased the relative recovery and thereby yielded lower glucose estimations than if we would have used the traditional rate at 0.3 µL/min. Because the variation in glucose and lactate estimations appeared larger for higher values, it is possible that a lower perfusion rate would translate into a slightly weaker correlation of the glucose and lactate estimations between Loke and standard MD. Furthermore, it is important to note that most established MD thresholds are based on a perfusion rate at 0.3 µL/min, and therefore Loke must be used at that rate if the previously established consensus thresholds are to be used [8].

Fourth, a last import note is that the standard Loke perfusion rate at 0.3 µL/min would yield a delay time between the catheter tip and the biosensors of close to 1 h. Thus, although the data frequency increases with Loke, the time until the data are available is comparable to standard CMD. Alternatively, rather than setting the exact same perfusion rate as the standard CMD to use the same metabolite thresholds (at least for glucose), it might be more appealing to increase the perfusion rate to decrease the delay time in clinical practice (i.e., similar to what was done in this study). This approach would definitely improve the clinical usefulness and timeliness of Loke, but it would make the translation between standard CMD and Loke MD estimations more complex.

By inducing variations in systemic and cerebral physiology, we were able to explore a large range of cerebral glucose and lactate with repeated measurements, despite a relatively small number of pigs. There were also some limitations of the study. As already outlined, the MD catheters were placed in adjacent brain tissue via two separate burr holes, with some risk of natural spatial variations in cerebral glucose/lactate for each catheter. In addition, different catheters were used for standard CMD (71 High Cut-Off Brain MD catheter) and Loke (the CMA 70 Brain MD catheter), but a previous study found that the estimations of energy metabolites are comparable for both catheters [30]. Furthermore, the standard MDs were not analyzed immediately but were instead stored in a freezer, which could have influenced the metabolite concentration to a smaller extent. In addition, for the purposes of our correlation analyses between Loke and standard MD, we adjusted the time lags based on catheter volumes and calculated mean values of continuous Loke MD to make their values more comparable in terms of time lag. Although this method did not depict the true chronology, it was the best way to capture the physiological chronology. In addition, according to the in vitro analyses conducted by M Dialysis (described in the technical manual by the company), the in vitro reliability was estimated to be 0.1mM or up to ± 30% for glucose and lactate. This could explain some of the variation between the methods but not the more pronounced difference between lactate estimations. Also, the study was quite short (around 4 h), and our results therefore mostly reflect the first hours after insertion, but it is possible that the quality of the electrochemical sensor changes over time in vivo. This is of relevance because neuromonitoring during acute brain injury often entails 3–7 days. Lastly, it would be of great interest to analyze cerebral pyruvate and to calculate the LPR; however, at present, this is not possible with the current biosensors.

## Conclusions

Continuous Loke MD showed a strong correlation with the absolute values and temporal changes as the standard CMD for glucose, but it appeared less reliable for high values. Although there was a significant correlation between Loke MD and standard CMD lactate, it was relatively weak, and the variation in absolute values was large both between and within each pig. Thus, it appears reasonable to use the same established MD glucose thresholds for Loke MD as for the standard MD with some caution, whereas this should be avoided for lactate. However, the optimal perfusion rate of Loke MD needs to be considered carefully because it affects the delay time between brain and sensor but also the comparability with previous studies using the standard intermittent MD technique. Lastly, Loke MD displayed a high short-term stability, indicating a high signal-to-noise ratio. This opens the door for high-frequency explorations of energy metabolism during brief and transient pathophysiological events, such as cortical spreading depolarizations. Altogether, Loke MD appears to be a promising tool for continuous monitoring of cerebral energy metabolism, but further explorations and validations of perfusion rates and thresholds are needed before clinical implementation

## Supplementary Information

Below is the link to the electronic supplementary material.

Supplementary file1 (DOCX 182 kb)

Supplementary file2 (DOCX 13 kb)
