# Supplementary material for: Preliminary Observations of the Loke Microdialysis in an Experimental Pig Model: Are We Ready for Continuous Monitoring of Brain Energy Metabolism?
Source: Neurocrit Care. 2024 Jul 31;42(1):222–31. doi: 10.1007/s12028-024-02080-5 (PMC11811243; doi:10.1007/s12028-024-02080-5)
Supplement: Supplementary file 1 — Supplementary file1 (DOCX 182 kb) [file 12028_2024_2080_MOESM1_ESM.docx]

**Supplementary figure 1. Experimental set-up – an example**


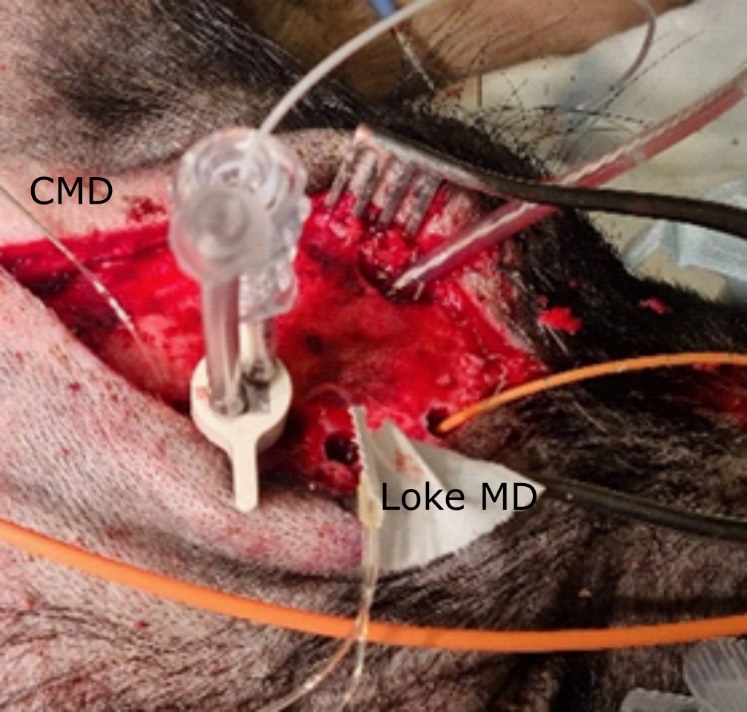


The figure shows the intubated and sedated pig with several multimodal monitoring tools in the right hemisphere and an intracranial balloon epidurally on the left side. The CMD and Loke MD are denoted in the figure.

CMD = Cerebral microdialysis.
