# Supplementary material for: Preliminary Observations of the Loke Microdialysis in an Experimental Pig Model: Are We Ready for Continuous Monitoring of Brain Energy Metabolism?
Source: Neurocrit Care. 2024 Jul 31;42(1):222–31. doi: 10.1007/s12028-024-02080-5 (PMC11811243; doi:10.1007/s12028-024-02080-5)
Supplement: Supplementary file 2 — Supplementary file2 (DOCX 13 kb) [file 12028_2024_2080_MOESM2_ESM.docx]

**Supplementary table 1. Pig characteristics**

| **Pig** | **Species** | **Sex (female/male)** | **Age (months)** | **Weight (kg)** |
| --- | --- | --- | --- | --- |
| 1 | Sus scrofa domesticus | Female | 2-3 | 34 |
| 2 | Sus scrofa domesticus | Female | 2-3 | 31 |
| 3 | Sus scrofa domesticus | Female | 2-3 | 35 |
| 4 | Sus scrofa domesticus | Male | 2-3 | 31 |
